# Schwann Cell-Derived Exosomal Vesicles: A Promising Therapy for the Injured Spinal Cord

**DOI:** 10.3390/ijms242417317

**Published:** 2023-12-10

**Authors:** Mousumi Ghosh, Damien D. Pearse

**Affiliations:** 1 The Miami Project to Cure Paralysis, University of Miami Miller School of Medicine, Miami, FL 33136, USA; mghosh@med.miami.edu; 2 The Department of Neurological Surgery, University of Miami Miller School of Medicine, Miami, FL 33136, USA; 3 Department of Veterans Affairs, Veterans Affairs Medical Center, Miami, FL 33136, USA; 4 The Interdisciplinary Stem Cell Institute, University of Miami Miller School of Medicine, Miami, FL 33136, USA; 5 The Neuroscience Program, University of Miami Miller School of Medicine, Miami, FL 33136, USA

**Keywords:** Schwann cells, exosomal vesicles, exosomes, spinal cord injury, neuroprotection, neurorepair

## Abstract

Exosomes are nanoscale-sized membrane vesicles released by cells into their extracellular milieu. Within these nanovesicles reside a multitude of bioactive molecules, which orchestrate essential biological processes, including cell differentiation, proliferation, and survival, in the recipient cells. These bioactive properties of exosomes render them a promising choice for therapeutic use in the realm of tissue regeneration and repair. Exosomes possess notable positive attributes, including a high bioavailability, inherent safety, and stability, as well as the capacity to be functionalized so that drugs or biological agents can be encapsulated within them or to have their surface modified with ligands and receptors to imbue them with selective cell or tissue targeting. Remarkably, their small size and capacity for receptor-mediated transcytosis enable exosomes to cross the blood–brain barrier (BBB) and access the central nervous system (CNS). Unlike cell-based therapies, exosomes present fewer ethical constraints in their collection and direct use as a therapeutic approach in the human body. These advantageous qualities underscore the vast potential of exosomes as a treatment option for neurological injuries and diseases, setting them apart from other cell-based biological agents. Considering the therapeutic potential of exosomes, the current review seeks to specifically examine an area of investigation that encompasses the development of Schwann cell (SC)-derived exosomal vesicles (SCEVs) as an approach to spinal cord injury (SCI) protection and repair. SCs, the myelinating glia of the peripheral nervous system, have a long history of demonstrated benefit in repair of the injured spinal cord and peripheral nerves when transplanted, including their recent advancement to clinical investigations for feasibility and safety in humans. This review delves into the potential of utilizing SCEVs as a therapy for SCI, explores promising engineering strategies to customize SCEVs for specific actions, and examines how SCEVs may offer unique clinical advantages over SC transplantation for repair of the injured spinal cord.

## 1. Introduction

Spinal cord injury (SCI) is a complex and debilitating condition that has a major health care cost and social burden. SCI leads to neuroinflammation, tissue loss, glial scarring, and the formation of a cyst that is associated with permanent loss of motor, sensory, and autonomic functions, and the antagonism of self-repair [1,2,3]. Despite considerable investigative research towards identifying new therapies, a cure for, or the significant restoration of lost function after, SCI remains elusive.

## 2. Spinal Cord Injury and Promising Therapeutic Approaches for SCI Repair

Promising therapeutic approaches that are currently in development broadly target the protection of nervous system tissue from further damage or promote axon regeneration, plasticity, and repair, to either lessen functional loss or restore function, respectively. These approaches include (1) pharmacological therapies that target the processes of inflammation, cell death, gliosis, or abortive axon growth [3,4,5,6]; (2) transplantation of cells into the injured cord to replace lost tissue or act as a bridge for regeneration [7,8,9,10,11], including Schwann cells [12,13,14,15], olfactory ensheathing cells [16,17,18], mesenchymal stem cells [19,20], or induced pluripotent stem cells [21,22,23,24]; (3) gene therapy with viral-vector-mediated delivery of genes encoding factors that are neurotrophic or neuroprotective [25,26,27,28,29]; (4) biomaterials and synthetic scaffolds of polymers and gels that can bridge the gap between injured axons and their targets or which possess the capacity to deliver growth promoting molecules [30,31,32,33,34,35]; (5) biologics, including the growth factors brain-derived neurotrophic factor (BDNF) and glial cell-derived neurotrophic factor (GDNF), that positively alter axon and cell responses towards repair [36,37,38,39]; (6) and electrical stimulation-based approaches, such as epidural stimulation (ES) with spinal cord-implanted electrodes to deliver electrical pulses [40] to improve motor function [41,42,43,44,45] or transdermal or transmagnetic electrical stimulation (TEM) to modulate activity after SCI [46,47].

These are just a few of the many promising therapeutic approaches currently under development for improving repair and recovery after SCI. By combining different approaches, researchers and clinicians are working to develop more robust therapies that can lead to significant functional recovery and improve the quality of life of SCI individuals [48,49,50,51,52,53,54,55,56].

## 3. Use of Schwann Cell Transplantation as a Promising Therapeutic Strategy for Injured Spinal Cord Repair

Schwann cells (SCs) are specialized myelinating glial cells of the peripheral nervous system (PNS) [57,58,59] that support and protect peripheral neurons as well as play a central role in successful PN regeneration. From a vast body of research in the last 30 years, SC transplantation (SCT) has been demonstrated to be a promising therapeutic approach for repairing the damaged nervous system, including SCI [60], peripheral nerve injury (PNI) [61,62,63], and multiple sclerosis (MS) [64,65]. SCT provides numerous benefits after SCI, such as limiting tissue damage, modulating inflammation [66], and reducing cystic cavitation as well as providing growth substrates and secreting growth factors for axon regeneration and remyelination repair, all of which collectively improve outcomes in experimental SCI models [67,68]. Phase 1 clinical trials have also been conducted to evaluate the safety and efficacy of SCT in acute and chronic human SCI [8,13,14,69]. Though the translation of SCT into clinical implementation, particularly as an autologous therapy, remains ongoing [12] and initial investigations have demonstrated its safety and feasibility [13,14], several challenges remain that limit its therapeutic efficacy [70]. The therapeutic potential of SCT [60] is restricted first by the invasive nature of intraspinal transplantation, as a surgery is required to enable the cells to be injected into the injured spinal cord. Another limitation is the substantial loss of the transplanted cells that occurs, with less than 20% of the transplanted SCs surviving weeks to months after injection [34,71], though the survival of SCs is superior to that of many other transplanted cell types, including bone marrow stromal cells (BMSCs) and stem cells [72]. SC survival is limited by cell shear stress during transplantation, mitogen withdrawal, and anoikis, as well as by their exposure to the harsh microenvironment of the injury site with hypoxia, excitotoxicity, inflammation, and immune cell-mediated rejection [73,74]. The importance of the 1:1 ratio of SCs to axons in their association means that the survival of the cells is very important for their repair function in guiding and myelinating axons. Transplanted SC survival can be enhanced by combining them with biomaterials [35] or anti-apoptotic agents [75,76], among other protective agents. Another limitation of SCT is their restricted integration and migration within the injured spinal cord following transplantation, which prevents them from guiding growing axons into and from the injury site as well as limits the ability of SCs to reach areas of distal demyelination. Altering the interaction of SCs with astrocytes by genetic modification of the cells to express surface polysialic acid (PSA) is one approach among several that allows transplanted SCs to migrate extensively within the injured spinal cord and support greater axon growth and functional recovery [29,77]. Lastly and specifically for cultured human SCs, a low capacity or inability to myelinate axons in SCI paradigms compared to rodent SCs may also negatively impact their therapeutic effectiveness [12,78]. Although numerous approaches are being investigated to overcome these deficiencies and improve the reparative efficacy of SCT after SCI, an alternative approach to SCT would be to derive from SCs therapeutic components that may exhibit some of the beneficial effects of the cells for neuroprotection and repair but do not possess these limitations. One such bioactive component is a small, nanosized secreted vesicle called the exosome (Table 1).

## 4. Exosomal Vesicles: A Next-Generation Cell-Derived Therapeutic Modality

Recent studies have highlighted the use of exosomal vesicles (EVs) as an alternative approach to cell therapeutics which may impart many of the same beneficial effects of the parent cells from which they are derived [79,80,99,100,101] (Table 1). EVs are nanoscale endocytic vesicles (30–150 nm) that are released into the immediate environment by cells as one way to allow for the transfer of functionally important biomolecules between them. Additionally, EVs contain a variety of cargos, from miRNA and proteins to signaling intermediaries that can promote cell survival, differentiation, and axon growth or subdue inflammation and alter scar formation in the recipient cells [82,87,102]. These nano-sized vesicles offer many advantages over classical cell therapy including (1) biocompatibility; (2) improved targeted delivery: exosomes can efficiently traverse the blood–brain or the blood–spinal cord barrier (BBB, BSCB) due to their small size and lipid bilayer composition [93]; (3) recipient cell targeting by receptor systems or plasma membrane fusion [103,104], which allows for more precise and effective delivery to target tissues or organs, potentially enhancing therapeutic outcomes; (4) minimal or no immunogenicity [94,105], a significant concern when using allogeneic cell transplants [106]; (5) specific molecular cargo: exosomes carry a cargo of bioactive molecules, including coding and noncoding RNAs, proteins, and nucleic acids, that can regulate cellular processes and modulate tissue repair to modulate cellular responses, thereby making them a versatile tool for therapeutic interventions [83]. Additionally, (6) EVs can be loaded to encapsulate drugs for delivery to delay drug elimination following systemic administration or (7) EV cargos can be tailored to address specific therapeutic goals, allowing for precise control over the treatment’s mechanism of action or can be selectively engineered with a variety of intracellular molecules and surface moieties [85,107], thereby allowing them to be attractive therapeutic carriers as drug delivery vehicles [95] and an alternative to cell therapeutic approaches for promoting repair [108,109,110,111]. (8) Unlike cell therapy, EVs pose a reduced risk of embolism: the smaller size of exosomes reduces the risk of vascular occlusion or an embolism, a potential complication associated with the infusion of larger-sized cells, which enhances the safety profile of EVs [92,112]; (9) EVs can be harvested and purified repeatedly from their parent cells and therefore are superior with respect to scalability and manufacturing than growing and expanding cells in culture, simplifying the production process and reducing costs [91,96]; (10) reduced risk of tumorigenicity: cell therapies using stem cells or progenitor cells may carry a risk of tumorigenicity, where the transplanted cells could potentially form tumors [113]. EV therapy largely avoids this risk because it involves only the administration of cell-derived vesicles with no capacity for self-replication or division.

Additionally, the ability to derive EVs from various cell types, including mesenchymal stem cells (MSCs), SCs, and immune cells [114,115,116], makes EVs highly versatile for customization for diverse medical conditions, including SCI. Overall, EVs present a promising avenue for precision medicine and regenerative therapies with several advantages over conventional cell-based approaches.

## 5. Current Status of Exosomal Vesicle Use as a Therapy in Spinal Cord Injury Repair

The use of EVs in SCI is an emerging and promising field of research and to date, few studies have assessed their therapeutic efficacy in experimental models. Preclinical studies have reported that EVs from a variety of cell sources can be beneficial in rodent models of SCI, providing improvements in histological and functional outcomes such as enhancing angiogenesis, stimulating axon regeneration or reducing inflammation [117,118,119,120]. The efficacy associated with EVs has been ascribed to their cargos, which include anti-inflammatory molecules, growth factors, and proregenerative or prosurvival microRNAs that stimulate endogenous cell activities, remodeling, and repair [99,121].

In studies employing intravenously delivered MSC-derived exosomes (MSCEVs), EVs were shown to target innate immune cells within the injured spinal cord [122], especially when administered acutely after SCI. Uptake of MSCEVs resulted in an increase in the numbers of CD206-expressing anti-inflammatory M2 macrophages within the injured spinal cord that were associated with a reparative phenotype. Mice receiving systemic infusion of these MSCEVs also exhibited improved locomotor performance in the Basso, Beattie, and Bresnahan (BBB) open-field locomotor test along with reductions in lesion size and increased formation of blood vessels in the injured segment compared to controls. MSCEV-treated animals exhibited reduced levels of proinflammatory cytokines TNF-α and IL-1β after SCI, and lower expression of proapoptotic proteins including Bcl2, suggesting that MSCEVs possess anti-inflammatory and neuroprotective activities [123]. Similarly, EVs derived from umbilical cord MSCs, when delivered via the tail vein in SCI rodents, led to improvements in behavioral performance and reduced cavitation at the injured segment, which was attributed to the ability of EVs to also induce the conversion of macrophages towards an anti-inflammatory phenotype [124,125,126]. Likewise, studies have demonstrated that the systemic administration of epidural fat-derived MSCEVs results in a decrease in injury size and enhanced recovery of neural function in rodent models of SCI, which occurred through the suppression of the activation of NLRP3 inflammasomes along with a reduction in the levels of inflammatory cytokines and proapoptotic factors [124]. In studies by Zhang and colleagues, bone marrow MSCEVs exhibited the capacity to promote improved sensory–motor function and spatial learning in a rat model of traumatic brain injury (TBI). The functional improvement in MSCEV-treated TBI groups correlated with an alleviation of cerebellar inflammation, increased neurogenesis in the hippocampal dentate gyrus, and an induction of neurovascular plasticity compared to controls [127]. Therefore, the collective results of these investigations point to the anti-inflammatory and protective activities of MSCEVs that allow them to perturb tissue damage and cell loss to thereby reduce neurological dysfunction after neurotrauma. These actions mirror similar benefits provided by the systemic delivery of MSCs themselves in injury paradigms; however, MSCEVs offer an approach that is more minimally invasive and without potential risk of tumorgenicity or embolism [73,74,128]. EVs derived from other cell sources have also shown benefit in models of SCI, including microglia-derived exosomal vesicles (MGEVs) that improved functional recovery by putatively reducing oxidative stress, improving endothelial cell survival, and promoting vascular regeneration in response to the SCI activation of the keap1/Nrf2/HO-1 signaling pathway [129].

## 6. Potential of Schwann Cell-Derived Exosomes (SCEVs) for SCI Repair

Recent work has shown that EVs derived from SCs (SCEVs) can markedly enhance axon regeneration in vitro as well as in vivo following PNI. While the critical role of SCs in endogenous PNI regeneration has been extensively documented, the involvement of SCEVs in this process has only emerged in the last decade [130,131,132]. Initial experiments using SCEVs expressing CD63-GFP revealed that the vesicles were taken up by axons [130]. Furthermore, SCEVs, when placed upon neurons, could accelerate axon regeneration both in in vitro cultures of dorsal root ganglion (DRG) neurons and in vivo following sciatic nerve injury [130]. Importantly, this effect of SCEVs was demonstrated to be specific to the cell type as EVs derived from primary fibroblasts did not elicit a similar regenerative response in neurons [130]. In subsequent investigations, it was found that only SCEVs released by ‘Repair SCs’ could promote axon regeneration after nerve injury [131] and that SCEVs from other SC phenotypes, such as ‘Differentiated SCs’ did not possess this behavior. These findings were also confirmed using in vitro cell-based assays with Differentiated and Repair SCs induced by high concentrations of phosphodiesterase-resistant cAMP analogues like CPT-cAMP or db-cAMP [133,134] or a low concentration of forskolin, respectively; the latter functions synergistically with neuregulin to drive the proliferating phenotype of SCs [135]. The axon-growth-promoting capacity of SCEVs from Repair SCs was attributed to the transference of miRNA-21 from SCEVs to axons, leading to the suppression of phosphatase and tensin homolog (PTEN) and the activation of phosphoinositide 3-kinase (PI3K) [131,136], molecular cascades implicated in the promotion of axon growth. The beneficial effects of SCEVs were replicated in SCs expressing c-Jun and Sox-2, characteristic markers of Repair SCs, and when c-Jun and Sox2 were silenced using lentiviral vectors, the axon-growth-promoting action of SCEVs derived from these cells was lost [131]. These studies highlight the importance of SCEVs and their cargos in SC–axon communication and axon growth as well as demonstrate a divergence in the beneficial effects provided by Repair versus Differentiated SCs on axon growth as mediated by SCEVs.

SCEVs are capable of modulating growth cone morphology and dynamics towards regeneration by inhibiting GTPase RhoA activity, which is involved in growth cone collapse and axon retraction [130]. The addition of SCEVs to axons was shown to reduce GTP-bound RhoA levels in growth cones. Moreover, DRGs exposed to SCEVs exhibited growth cones with significantly more filopodia, which is an indicative marker of RhoA suppression, and elevated actin polymerization within growth cones [130,137]. This proregenerative mechanism induced by SCEVs offers an explanation for their therapeutic action in promoting axon growth in vitro and after PNI.

Proteomic analysis of SCEVs has identified that they contain more than 430 proteins. Of these, 398 proteins were found to match those in the ExoCarta database, with 12 being particularly pertinent to CNS repair. These include carboxypeptidase E (CPE), fatty acid-binding protein (FABP5), fibronectin, flotillin-2, major vault protein (MVP), monocarboxylate transporter 1 (MCT1), neuropilin-2 (NRP2), septin-7 (SEPT7), protein disulfide-isomerase A3 (PDIA3), and syntenin-1 [138]. Similarly, proteins such as αB-crystallin and galectin-1 identified in SCEVs have been linked to potential anti-inflammatory actions [138]. Biological pathway analysis has revealed a connection between some of the prominent pathways associated with proteins in SCEVs and crucial signaling pathways like MAPK, PI3K-Akt, and cAMP signaling, all of which play significant roles in CNS regeneration and repair [138]. Interestingly, SCEVs exhibit an enrichment of certain proteins in the ribosome pathway, which provides additional evidence to the crucial role that SCs play in facilitating protein synthesis within axons. SCEVs also contain members of the Rho GTPase family, including Rac1 and Cdc42, which are associated with neuronal development, survival, and the facilitation of neurite growth and regeneration [139,140]. These GTPases also play a role in promoting the migration of oligodendrocytes, crucial cells for CNS myelination [141], and contribute to improved outcomes after SCI by preserving BSCB integrity and protecting endothelial cells [142].

**Scheme 1:**
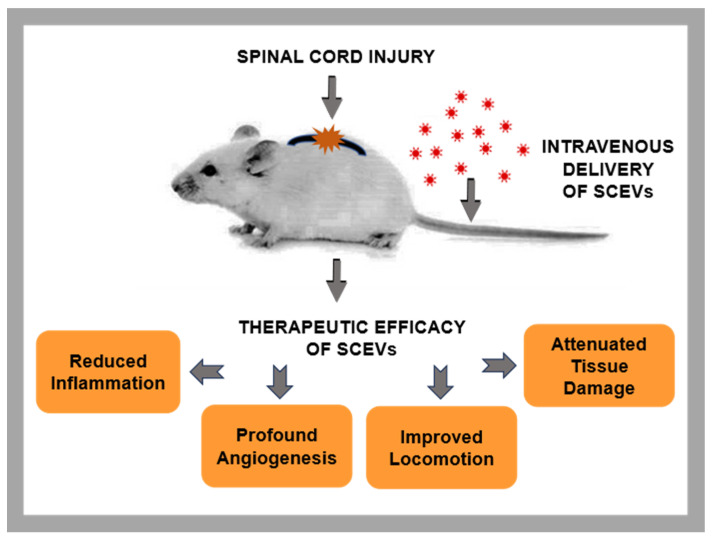
Therapeutic benefits of administering SCEVs in rodent models of SCI.

Following these initial investigations that established the importance of SCEVs in axon growth and PNI regeneration in vitro, subsequent work has assessed whether SCEVs could be beneficial in models of SCI (Scheme 1). Delivery of SCEVs to SCI adult male Sprague-Dawley rats confirmed their capacity to provide neuroprotection and improved functional recovery. In these studies, SCEV cargo analysis indicated that integrin-β1 was one of the main constituents and likely played a crucial role in promoting angiogenesis after SCI. This was confirmed both by immunohistological assessments as well as experiments using shRNA-mediated inhibition of integrin-β1 expression in the SCEVs that suppressed their angiogenic effects on endothelial cells [116]. In subsequent work, proteomic profiling of SCEVs revealed several proangiogenic molecules, including integrins and VEGF, that were highly expressed in SCEVs. SCEV integrin-β1 and integrin-α1 were found to contribute to angiogenesis after SCI and aid in attenuating tissue damage, reducing lesion size, and improving function compared to controls.

Studies by Ren et al. [143] have investigated the effects of SCEVs on the inflammatory response after SCI contusion in rodents. Tail vein administration of SCEVs enhanced functional recovery, as demonstrated by significantly higher BBB scores. Electrophysiological assessment in SCI animals exposed to SCEVs showed an improvement in motor evoked potentials (MEPs), suggesting an improvement in nerve conduction. The SCI + SCEV group was associated with shortened MEP latency and increased amplitude suggesting a SCEV-driven improvement in functional output. Likewise, catwalk gait analysis of SCEV-treated SCI rats showed a restoration of the regularity index, improvements in hind limb stance, rear limb coordination, and increased limb supporting timings. Histopathological assessment of the injured spinal cord showed that the lesion was significantly reduced following treatment with SCEVs. The bladders of SCEV-treated animals showed reduced thickness along with a more rapid recovery of bladder function in the treatment group. Immunohistological evaluation of spinal cord tissue at 28 days post injury showed a reduction in the inflammatory response that was associated with modulation of macrophage/microglia cells towards M2 polarization along with a concurrent reduction in neuronal apoptosis. Quantitative analysis of ED1 + immune cells showed a reduction in iNOS and increased CD206 expression in the spinal cord encompassing the site of injury after SCEV delivery that correlated with an increased intensity of Neurofilament (NF) 200 fluorescence and numbers of choline acetyltransferase (ChAT)-positive neurons, suggestive of increased neuronal preservation.

Proteomic profiling of SCEVs has revealed that milk fat globule-epidermal growth factor-factor 8 (MFG-E8) is a key cargo component that is found in high amounts in SCEVs. MFG-E8 is thought to be a major contributor to their anti-inflammatory action after SCI through its ability to promote immune modulation. Prior work demonstrated the neuroprotective role of MFG-E8 in various neurodegenerative conditions including TBI [42], primarily by attenuating proinflammatory cytokine production and reprogramming microglia towards a neuroreparative phenotype. The protective role of SCEV-derived MFG-E8 was shown using SCEVs derived from lentiviral-mediated SC knockout cells of MFG-E8 (shMFG-E8-KO), in which the anti-inflammatory activity of SCEVs was lost post SCI. Likewise, in vitro experiments with shMFG-E8-KO SCEVs demonstrated that they were unable to promote M2 polarization of LPS-stimulated BMDMs expressing high levels of iNOS and CD86. It is unclear, however, whether the SCEVs used in these studies were able to induce neural proliferation and differentiation or promote axon growth following SCI as these outcomes were not assessed.

Similar to the proregenerative and anti-inflammatory properties of SCEVs derived from primary SCs, SCEVs obtained from skin precursor-derived SCs (rat SKP-SCs) can also be internalized by neurons in vitro and in vivo and have been demonstrated to enhance axon growth. Experiments employing an in vitro model of nerve axotomy and an in vivo nerve crush injury paradigm of motoneurons showed that both responded with enhanced axon growth in the presence of SKP-SCEVs. Similar results were also obtained in a rat ischemic–hypoxic injury model of motoneuron exposure to oxygen–glucose deprivation (OGD), where treatment with SKP-SCs augmented neuronal viability and axon growth. Subsequent experiments implicated the Akt/mTOR/p70S6K pathway as the primary signaling mechanism underlying the proregenerative and prosurvival effects of SKP-SCEVs in these motor neuron injury models, with the effectiveness of SKP-SCEVs being suppressed by rapamycin. These studies highlight the potential of SKP-SCEVs not only in models of CNS injury but also motoneuron diseases like ALS [144].

SCEVs have also shown the ability to mitigate mitochondrial dysfunction and necroptosis, both in vivo in rodent SCI compression and in vitro in rat pheochromocytoma cells (PC12) exposed to OGD [145]. Treatment with SCEVs was shown to reduce oxidative stress and attenuate inflammatory responses following SCI, while also alleviating necroptosis. Similarly, SCEVs improved mitochondrial functionality and prevented the formation of necrotic cells. Injured, SCEV-treated PC12 cells showed enhanced mitophagy, a critical process necessary for maintaining proper cellular functions [146], which in turn was associated with the inhibition of ROS generation and inflammatory cytokine expression after OGD. SCEVs also attenuated mitochondrial dysfunction and necroptosis in this paradigm. Although the precise mechanism by which SCEVs alleviate mitochondrial dysfunction after SCI is unclear, subsequent investigations indicated that SCEVs possessed the ability to stimulate the activation of AMP-activated protein kinase (AMPK), a signaling pathway that is known to be associated with mitochondrial autophagy [146], oxidative stress attenuation, and reduced inflammation.

Of note, the mechanical stimulation of SCs has been reported to generate SCEVs with a greater capacity for inducing neurite outgrowth and in vivo peripheral nerve regeneration compared to naïve SCEVs. The use of miRNA sequencing of these mechanically-stimulated SCEVs has identified miR-23b-3p as a key factor responsible for driving axon regeneration, while neuropilin 1 was determined to be the target gene in neurons. SCEVs from mechanically stimulated SCs were found to have decreased neuronal Nrp1 expression that was implied to be involved in their proregenerative effects [147].

SCEVs from healthy donor SCs have been employed to treat diabetic peripheral neuropathy (DPN). Intravenous SCEV delivery to type 2 diabetic db/db mice suffering from peripheral neuropathy significantly improved sciatic nerve conduction velocities and increased sensitivity to thermal and mechanical stimuli as well as increased the remyelination of sciatic nerves. Further analysis revealed that SCEVs were able to reverse the diabetes-induced reduction in several essential miRNAs, such as miR-21, -27a, and -146a, along with the modulation of a diabetes-induced elevation in semaphorin 6A (SEMA6A), RhoA, PTEN, and nuclear factor-kappa B (NF-kB) [148].

In work by Pan et al. (2021), SCEVs were used in a mouse severe crush model of SCI. SCEVs produced a substantial increase in TLR2 expression in astrocytes with a concomitant reduction in the deposition of CSPGs as well as enhanced functional recovery. Notably, the targeted knockout of TLR2 in astrocytes reversed the SCEV-induced decrease in CSPG deposition that occurred through alterations in NF-κB/PI3K signaling, inhibiting functional recovery post SCI [149]. In a subsequent study by Pan et al. (2022), tail-vein-delivered SCEVs administered thrice per week for a period of one month beginning on the day of the SCI promoted repair by enhancing autophagy while reducing apoptosis. This dual action of SCEVs mitigated SCI-induced axon damage and restored motor function, a behavior that was mediated through effects on the EGFR/Akt/mTOR signaling pathway [150].

**Scheme 2:**
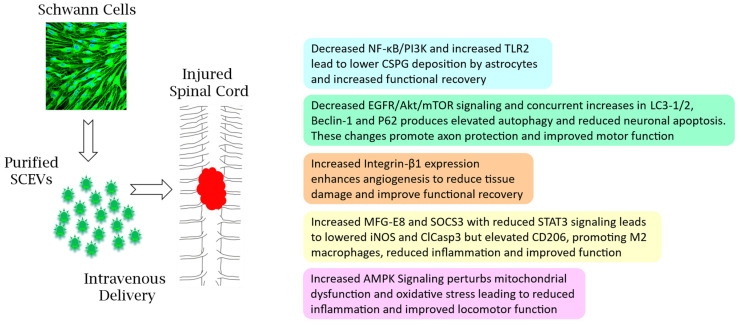
Alterations in signaling pathways linked to the intravenous administration of SCEV and correlated to both histological and functional benefits of SCEVs in rodent SCI models.

In a recent study, Zhu and colleagues [151] developed a noninvasive nanofiber scaffold containing a hyaluronic acid hydrogel patch. This patch was designed to release both SCEVs and methylprednisolone to the injured spinal cord. The composite drug–EV patch demonstrated favorable biocompatibility, maintaining SCEV morphology and exhibiting low toxicity to nerve cells. The in vivo application of the composite patch on the hematoma surface significantly enhanced the functional and electrophysiological performance of SCI rats. Additionally, the composite patch resulted in a suppressed inflammatory response by inducing a shift in macrophage polarization from the M1 to M2 phenotype as well as enhanced neuronal survival by inhibiting apoptosis after SCI, an effect that was ascribed to the modulation of the TLR4/NF-κB, MAPK, and Akt/mTOR pathways. This combinatorial drug–SCEV patch therapy thereby appears to significantly improve the efficacy of SCEVs for SCI repair. Scheme 2 shows the different signaling mechanisms that are associated with the intravenous administration of SCEV and the histological and functional benefits provided after experimental SCI.

## 7. Limitations of SCEVs as a Therapeutic Modality for Nervous System Repair

Mature SCs have been shown to exhibit remarkable phenotypic plasticity, possessing the capacity to transition between multiple functional states in response to environmental cues, including to a proliferating or immature state [152] that can in turn undergo differentiation into myelinating or nonmyelinating (Remak) forms [153] or reprogrammed to a Repair SC state in response to nerve injury [154]. Each SC form retains the ability to switch between phenotypes in response to environmental signals and appropriate cues [152]. While the different SC phenotypes can provide a broad range of functions that can contribute to the protection and repair of the nervous system, specific SC states are also associated with the initiation of pathological processes, including neuropathies, chronic pain [155], and tumorigenesis [156,157]. The distinction between the beneficial and pathological actions of the different phenotypes of SCs appears to be related to the EVs they produce, and more specifically, the microRNA cargos these SCEVs carry. Recent investigations have shown that distinct phenotypes of SCs release EVs with unique cargos that confer neuroprotection, are proreparative, or which influence axonal conduction and synaptic transmission [62,130,131,143] or play a role in axon myelination [158], or, conversely, trigger the development of peripheral neuropathies [159,160].

With the use of next-generation sequencing (NGS) arrays, profound differences in the miRNA content of SCEVs derived from cyclic AMP-differentiated phenotypes of SCs compared to normal SCs were identified [161]. SCEVs from differentiated SCs potently suppressed cell migration and proliferation, displaying alterations in miRNA content that differed from SCEVs derived from undifferentiated SCs. Cargo assessment of the SCEVs derived from cAMP-treated differentiated SCs showed increased levels of miR211 and miR92a-3p, along with decreased hypoxia-inducible factor (HIF), cyclin-dependent kinase 2 (CDK2), and platelet-derived growth factor C (PDGFc), correlating with corresponding phenotypic distinctions from control SCs [161]. These studies demonstrate that differences in the functional output of disparate SC phenotypes are largely driven by differences in the molecular cargos of the SCEVs they produce.

Apart from the distinctive roles of SCEVs in mediating the functional effects of different SC phenotypes, SCEVs have been reported to be central to pathological conditions and exert detrimental activities, such as inducing peripheral neuropathies after PNI. These pathogenic SCEVs appear to contain specific cargos that result from genetic or metabolic dysfunction in the SCs from which they arise. In work by Jia et al. [162], it was shown that SCEVs derived from SCs stimulated with elevated concentrations of glucose (HG-stimulated SCs) contributed to the development of diabetic peripheral neuropathy (DPN) in BKS.Cg-m+/+Leprdb/J (db/db) animals, a rodent model with diabetes exhibiting mechanic and thermal hypoesthesia and an impairment of nerve conduction velocity. SCEVs from HG-stimulated SCs inhibited the growth of distal axons and were found to contain increased levels of miRNA-28, miRNA-31a, and miRNA-130a, which altered levels of target proteins including DNA methyltransferase-3a, NUMB endocytic adaptor protein, synaptosome-associated protein 25 (SNAP25), and growth-associated protein 43 (GAP43) within axons. SCEVs caused metabolic dysfunction through increased polyol activity, impaired mitochondrial function, and elevated oxidative stress [162]. Reports have also postulated a role of SCEVs in Schwannomatosis, where dysfunctional SCEVs are released from injured Repair SCs due to a chronic insult from the tumors. The altered SCEVs in Schwannomatosis appear to exert a pathogenic influence on remote cells, potentially contributing to the formation of premetastatic niches.

It is therefore increasingly evident that SCEVs, while possessing great therapeutic potential, can also exert pathogenic effects according to the state of the SCs from which they are derived and the molecular cargos they carry [152]. While further research is necessary to advance the clinical utilization of SCEVs, recent advancements have clearly demonstrated that the versatility of SCEVs is heavily influenced by the phenotypic plasticity of SCs and that understanding and modifying the state of the parent cell from which they are derived will be critical to maximizing their therapeutic efficacy while ensuring that they do not exert pathological actions.

## 8. Engineering Schwann Cell-Derived Exosomes for Maximizing Therapeutic Efficacy

SCEV engineering using genetic and chemical techniques can be employed to enhance targeted drug delivery, offering multiple benefits including increased availability and a localized concentration of therapeutic agents, while minimizing potential drug-related side effects [163]. SCEV surface moieties can be altered to achieve cell- or tissue-selective targeting [164,165]. EVs have been engineered to conjugate fluorescent proteins to the EV surface for visual tracking and imaging studies, such as monitoring their release or uptake by recipient cells or after in vivo administration [166]. With genetic approaches, a targeting molecule is fused with an EV membrane protein at the gene level, which is subsequently overexpressed within the donor cells so that the produced EVs contain the targeted molecule of interest. Genetic engineering of EVs provides a readily accessible means for the incorporation of target peptides, proteins, or antibodies on the EV surface but necessitates the construction of plasmids and their overexpression in the donor cells [163]. One commonly employed approach has been the use of the N-terminus of LAMP-2B (lysosome-associated membrane protein 2B) which constitutes a large N-terminal extramembrane domain, a C-terminal transmembrane region, and a short cytoplasmic distal end, which is expressed on the surface of EVs [163,167]. Donor cells transfected with a plasmid containing the targeting sequence of interest fused to the extracellular domain of LAMP-2B at the N-terminus will then generate EVs decorated with the engineered peptide or protein ligands. Another example of genetic engineering of EVs has been accomplished using the rabies virus glycoprotein (RVG) peptide (TIWMPENPRPGTPCDIFTNSRGKRASNG), which confers selective affinity of EVs to neurons via binding to acetylcholine receptors and has been employed for the administration of drugs to the CNS [168,169]. Despite the advantages of engineering EVs by genetic approaches, EVs with surface-displayed peptides may still be susceptible to degradation. This issue has been overcome by adding a glycosylation motif to the N-terminus of peptide-LAMP-2B fusions [170]. Other transmembrane proteins, such as protein platelet-derived growth factor receptor (PDGFR) or the tetraspanin family of EV proteins CD63/CD9/CD81, or signal peptides such as the 37-residue glycosylphosphatidylinositol (GPI) signal peptide DAF or palmitoylation signal peptide (MLCCMRRTKQ), can similarly be engineered for surface display of targeting proteins or peptides of interest on EVs [163,171].

Similar to genetic engineering, the EV surface can also be altered using chemical methods in which conjugation reactions involving covalent modifications can be employed to stably modify EV surface proteins following binding to both small molecules and macromolecules. The amine groups found in EV proteins can be readily modified by attaching alkyne groups. Subsequently, these alkyne-labeled EV proteins can be linked to azide-containing compounds through copper-catalyzed azide-alkyne cycloaddition (CuAAC) reactions (click chemistry) to attach proteins or peptides on the EV membrane surface [172]. Chemical approaches, however, may result in the inactivation or aggregation of the target proteins or affect the stability of EVs. Despite some disadvantages associated with genetic and chemical approaches for modifying EV surfaces, both methods have been employed across many research labs to improve the effectiveness of EVs as a therapeutic modality.

Lastly, EVs can also be engineered for encapsulation in a biomaterial scaffold to further optimize and regulate drug release during in vivo administration, permitting slower EV release or increasing protection or stability to extend the circulation half-life of EVs after systemic delivery. The combination of biomaterials with EVs aids in shielding the surface-exposed proteins from proteolytic degradation and helps overcome their limitations of short circulation and rapid clearance in vivo [97,163,173]. Similarly, the combination of EVs with inorganic metal-based nanoparticles as imaging probes such as iron oxide nanoparticles or gold (Au) nanoparticles allows for both long-term and enhanced-contrast imaging for diagnostic purposes, especially for in vivo tracking of the localization and biodistribution of systemically injected EVs [81,174].

## 9. Future Prospectives and Limitations of Schwann Cell-Derived Exosomes in Spinal Cord Injury Repair

Exosomes derived from Schwann cells exhibit unique advantages for SCI repair when compared to those from other cell types. SCs, specifically, play a crucial role in the peripheral nervous system and possess intrinsic regenerative capabilities. Several studies have demonstrated the potential of SCEVs as a therapeutic avenue for treating SCI (Table 2), showing that SCEVs are capable of favorably modulating neuroinflammation, providing tissue protection, and stimulating axon growth and myelination. SCEVs thus present a promising avenue for SCI repair due to their inherent neurosupportive properties, anti-inflammatory properties, myelin repair, regenerative potential, and biocompatibility with the nervous system. These distinctive characteristics make them a preferred choice for therapeutic applications aimed at promoting recovery and regeneration in SCI.

However, the field remains in its infancy and many questions remain regarding how to optimize and translate this approach into the clinical setting as well as determine what safety concerns exist with SCEVs in different paradigms. In further development of SCEVs, it will be important to understand which cell culture conditions for SCs lead to the production of the most favorable vesicles and how to obtain SCEVs in high yield for potential repetitive dosing strategies.

Another significant hurdle in utilizing exosomes for therapeutic purposes is the absence of standardized protocols for isolating and purifying them. Currently, diverse methods are utilized to obtain purified exosomal populations from various donor cell types, including ultracentrifugation, ultrafiltration, size exclusion chromatography, polymer precipitation, and immunoaffinity. These methods are commonly employed in different laboratory settings. Each approach comes with its set of advantages and limitations, influencing critical factors such as yield, purity, exosomal integrity, cargo, presence of protein contaminants, formation of aggregates, and the cost, time, and labor intensity of the purification process [175]. Moreover, both single-stranded and double-stranded DNA has been identified in association with extracellular vesicles (EVs) that may possess tumorigenic potential. Hence, it is crucial to consider the removal or treatment of SCEVs using S1 nucleases, DNAse 1, or Exonuclease 111 to break down single-stranded DNA (ssDNA) or double-stranded DNA (dsDNA) [176]. This step is essential for eliminating any tumorigenic potential of SCEVs before their utilization for clinical studies. Additionally, the generation of large banks of autologous SCEV for clinical applications is neither cost- nor time-effective and poses a major hurdle to treating the donor SCI patient if SCEVs from allogenic individuals exhibit unfavorable immunogenic properties. Therefore, a comprehensive understanding and unified standardization of methods for the isolation, purification, analysis, and processing of SCEVs is critical in paving the way for the practical application of SCEVs in the clinical setting.

For preclinical testing, questions remain on what dosing regimens are optimal, how best to target the uptake of SCEVs to the nervous system when delivered systemically and whether SCEVs hold potential for improving outcomes in the most prevalent form of SCI, chronic injuries. Engineering SCEVs for the delivery of drugs, biologics, or labeling agents for tracking holds significant promise for obtaining a greater therapeutic benefit and broader utility but further optimization of methods for incorporating these agents onto or within SCEVs remains to be undertaken. In contrast to those endeavors focused on optimizing the therapeutic potential of SCEVs, it will also be important to investigate putative safety issues and other limitations associated with the approach. These include understanding how SCEVs alter different components of the immune system and whether differentially generated or engineered SCEVs exhibit increased immunogenicity and determining if toxicity exists with either high or long-term dosing of SCEVs in vivo. Another key challenge lies in the standardization of SCEV isolation and characterization methods. Variability in EV content and quality among different preparations could impact the reliability of therapeutic outcomes. In conclusion, SCEVs offer exciting prospects for SCI treatment and a promising future awaits their use in neurological applications for injury and disease. Improving our understanding of the mechanisms of their therapeutic effects and optimizing protocols for SCEV generation, characterization, and engineering will undoubtedly provide SCEVs that are more effective for SCI protection and repair in moving towards future clinical implementation.

## Figures and Tables

**Table 1:** Advantages and disadvantages of SCEVs as a therapy versus their parent cells.

Aspect	SCEVs Benefit over SCs	SCEVs Limitations to SCs	Ref.
Delivery method	Ease of systemic administration; delivery of EVs is minimally invasive and amenable to repetitive dosing.		[79,80,81]
Crossing barrier	Smaller-size SCEVs will make it easier to overcome cell barriers and diffuse in the extracellular space. EVs can effectively cross the blood–brain or the blood–spinal cord barrier.		[82]
Targeted effect	The ability to engineer SCEV surfaces to design targeted delivery of specific protein or RNA cargo will allow for cell-specific targeting.	Ambiguity in identifying targeted recipient cells. Mechanism of SCEV recognition and specific uptake by target cells is unclear.	[83,84,85,86]
Immunogenicity	Inherent advantages of EVs in terms of immune evasion will eliminate the need of using immune suppression following administration.		[73,87]
Circulation time and half-life		Short circulation time following systemic administration (mins to an hour) due to rapid removal by the immune system, which may result in only a transient biological effect. May require repeated dosing for persistent or long-term effect.	[86]
Zeta potential		Poor zeta potential, can lead to formation of unstable EV aggregates that may minimize efficacy, interfere with delivery, hinder with identification of optimal dosing, reduce circulation time, and induce an immune response.	[88]
Storage and shelf life	Long-term and simpler storage and shipping required compared to parent cells.	Alterations in the cargo consistency of EVs due to outward leakage of cargo content during storage.	[89]
Manufacturing challenges		Additional processing steps required beyond cell isolation and culture. Yield limited by growth conditions and characteristics of parent cell. Lack of standardized protocols for isolating and purifying SCEVs.	[89]
Cost		Scalability and manufacturing greater than parent cell, increasing production cost.	[90]
Safety concerns	Enhanced safety profile. Lower tumorgenicity potential. Reduced risk of embolism.		[91,92]
Tool for therapeutic interventions	Versatile molecular cargo of coding and noncoding RNAs, proteins, and nucleic acids that can regulate cellular processes and mediate tissue repair.		[93,94,95,96,97]
Intercellular interactions		Lack of direct cell–axon association and guidance properties. Cannot directly replace lost neural cells. Cannot provide a structural substrate for repair like parent cells.	[60,98]
Regulatory approval	Associated with fewer ethical constraints compared to the therapeutic use of parent cells in people.		[79]

**Table 2:** Comparative summary of studies on the effects of SCEVs in experimental models of SCI.

SCI Model	SCEV Purification	EV Delivery	Dose	Outcomes	Reference
Mouse, level: T10, severe spinal cord crush injury.	Centrifugation: 1000× *g* for 10 min 10,000× *g* for 30 min Ultracentrifugation: 100,000× *g* for 1 h Washing with PBS buffer 100,000× *g* for 1 h	Three administrations a week for 4 weeks via tail vein injection after induction of SCI.	250 µL of 0.1 μg/μL	Functional recovery accompanied by reduced CSPG deposition.	Pan et al., 2021 [149]
Rat, level: T10, spinal cord contusion. Injury induced using fall of a 10 g rod from a height of 2.5 cm.	Centrifugation: 1000× *g* for 10 min 10,000× *g* for 30 min Ultracentrifugation: 100,000× *g* for 1 h Washing with PBS buffer 100,000× *g* for 1 h	Three administrations a week for 4 weeks via tail vein injection after induction of SCI.	250 µL of 0.1 μg/μL	Increased autophagy, reduced apoptosis, decreased injury cavitation, axonal protection, and improved recovery of motor function.	Pan et al., 2022 [150]
Rat, level: T10, spinal cord contusion. Injury induced using fall of a 8 g rod from a height of 4 cm.	Centrifugation: 1000× *g* for 10 min 1000× *g* for 20 min Filtration using a 0.22 μm filter Ultracentrifugation: 100,000× *g* for 70 min	Single administration via tail vein injection initiated at 30 min post SCI.	100 µg	Attenuated tissue damage, reduced lesion size. Profound angiogenesis and improved BBB scores after SCI.	Huang et al., 2023 [116]
Rat, level: T9, spinal cord clip compression. Injury induced using 30 g force using a vascular clip.	Centrifugation: 300× *g* for 5 min 2000× *g* for 20 min 10,000× *g* for 60 min Filtration using a 0.22 μm filter Ultracentrifugation: 110,000× *g* for 70 min	Daily administration via tail vein injection during the initial week after SCI followed by three injections per week until survival endpoint.	500 μL of 0.1 mg	Reduced inflammation by alleviating oxidative stress. Increased mitophagy to minimize mitochondrial dysfunction. Reduced axonal damage and improvement in BBB scores.	Xu et al., 2023 [145]
Rat, level: T10, spinal cord contusion. Injury induced using fall of a 10 g rod from a height of 2.5 cm.	Centrifugation: 300× *g* for 5 min 2000× *g* for 20 min 10,000× *g* for 60 min Filtration using a 0.22 μm filter Ultracentrifugation: 130,000× *g* for 70 min	Three administrations per week via tail vein injection, initiated at 30 min post SCI.	50 µL (0.1 μg/μL)	Inhibited neuronal apoptosis, reduced inflammation, promoted M2 macrophage polarization, improved nerve conduction, bladder function, and BBB scores after SCI.	Ren et al., 2023 [143]
